# Blood-based lung cancer biomarkers identified through proteomic discovery in cancer tissues, cell lines and conditioned medium

**DOI:** 10.1186/s12014-015-9090-9

**Published:** 2015-07-16

**Authors:** Charles E. Birse, Robert J. Lagier, William FitzHugh, Harvey I. Pass, William N. Rom, Eric S. Edell, Aaron O. Bungum, Fabien Maldonado, James R. Jett, Mehdi Mesri, Erin Sult, Elizabeth Joseloff, Aiqun Li, Jenny Heidbrink, Gulshan Dhariwal, Chad Danis, Jennifer L. Tomic, Robert J. Bruce, Paul A. Moore, Tao He, Marcia E. Lewis, Steve M. Ruben

**Affiliations:** Celera employees during the course of these studies, Celera, 1311 Harbor Bay Parkway, Alameda, CA 94502 USA; Department of Cardiothoracic Surgery, NYU Langone Medical Center, 530 First Avenue, New York, NY USA; Division of Pulmonary, Critical Care, and Sleep Medicine, NYU School of Medicine, New York, NY USA; Division of Pulmonary and Critical Care Medicine, Mayo Clinic, Rochester, MN USA; Division of Oncology, National Jewish Health, Denver, CO USA

**Keywords:** Lung cancer, Early detection, Biomarker, Mass spectrometry, Proteomics, Discovery

## Abstract

**Background:**

Support for early detection of lung cancer has emerged from the National Lung Screening Trial (NLST), in which low-dose computed tomography (LDCT) screening reduced lung cancer mortality by 20 % relative to chest x-ray. The US Preventive Services Task Force (USPSTF) recently recommended annual screening for the high-risk population, concluding that the benefits (life years gained) outweighed harms (false positive findings, abortive biopsy/surgery, radiation exposure). In making their recommendation, the USPSTF noted that the moderate net benefit of screening was dependent on the resolution of most false-positive results without invasive procedures. Circulating biomarkers may serve as a valuable adjunctive tool to imaging.

**Results:**

We developed a broad-based proteomics discovery program, integrating liquid chromatography/mass spectrometry (LC/MS) analyses of freshly resected lung tumor specimens (*n* = 13), lung cancer cell lines (*n* = 17), and conditioned media collected from tumor cell lines (*n* = 7). To enrich for biomarkers likely to be found at elevated levels in the peripheral circulation of lung cancer patients, proteins were prioritized based on predicted subcellular localization (secreted, cell-membrane associated) and differential expression in disease samples. 179 candidate biomarkers were identified. Several markers selected for further validation showed elevated levels in serum collected from subjects with stage I NSCLC (*n* = 94), relative to healthy smoker controls (*n* = 189). An 8-marker model was developed (TFPI, MDK, OPN, MMP2, TIMP1, CEA, CYFRA 21–1, SCC) which accurately distinguished subjects with lung cancer (*n* = 50) from high risk smokers (*n* = 50) in an independent validation study (AUC = 0.775).

**Conclusions:**

Integrating biomarker discovery from multiple sample types (fresh tissue, cell lines and conditioned medium) has resulted in a diverse repertoire of candidate biomarkers. This unique collection of biomarkers may have clinical utility in lung cancer detection and diagnoses.

**Electronic supplementary material:**

The online version of this article (doi:10.1186/s12014-015-9090-9) contains supplementary material, which is available to authorized users.

## Background

Lung cancer is the leading cause of cancer mortality in the United States. Estimates for 2014 indicate that 224,210 individuals will be diagnosed with lung cancer and 159,260 will die from the disease [1]. The average 5-year survival is about 17 %, with 79 % of cases being diagnosed as regional or distant disease. If lung cancer is detected when localized, survival increases to over 50 % [1].

Support for early lung cancer detection has emerged from the landmark NLST, where LDCT screening was shown to confer a 20 % reduction in lung cancer mortality in a high risk population [2]. Despite concerns associated with the low specificity (73.4 %) of CT screening [3] and the resulting large number of false-positive findings for lung cancer (96.4 %), the USPSTF recently recommended annual LDCT-screening for lung cancer in high-risk individuals [4, 5]. In their recommendation statement, the USPSTF stressed the need for more research into the use of biomarkers to complement LDCT screening. Two key clinical opportunities exist. First, the use of biomarkers for early detection of lung cancer could define a new high-risk population or refine the screening criteria recommended by USPSTF (age: 55 to 80 years, smoking history: >30 pack-year). Such biomarkers would serve as a pre-imaging filter, reducing the overall cost of screening and lowering the number of false-positive findings and unnecessary follow-up procedures. The second opportunity lies in improving the accuracy of lung cancer diagnosis. Given the high frequency of positive findings (pulmonary nodules) with CT screening [2], new means of accurately determining malignant risk are urgently required. In the NLST, 24 % of surgically resected nodules were found to be benign [2]. By improving the accuracy with which malignant risk is determined, biomarkers could potentially enhance diagnostic management by reducing unnecessary surgical intervention, minimizing the use of costly PET-CT and lowering radiation exposure associated with CT monitoring, while enabling detection of lung cancer at an early, more curable, stage.

A wide variety of approaches have been utilized to discover new blood-based lung cancer protein biomarkers [6]. These range from splice variant analysis and the isolation of tumor-enriched transcripts [7], to the development of novel proteomic platforms with the capacity to resolve candidate markers in a highly multiplexed fashion [8]. Advances in mass spectrometry (MS)-based technologies have also enabled discovery of new lung cancer biomarker candidates directly in serum or plasma [9–13]. While the identification of biomarkers directly in blood-based matrices can be problematic due to their complexity and the presence of multiple highly abundant factors [14], some of these challenges can be minimized through extensive fractionation [15]. Differentially expressed candidate markers have also been successfully identified through comparison of blood draining from the tumor vascular bed matched with systemic arterial blood from the same patient [16].

Alternative, “indirect” MS approaches have also been successfully employed, wherein candidate markers initially identified in lung cancer tissue specimens, cell lines or conditioned medium, have subsequently been shown to be differentially expressed in the peripheral circulation using immunoassay-based methodologies. Pioneering discovery studies employed conditioned medium derived from the lung cancer cell line A549 or cell and organ cultures, followed by confirmation of expression profiles in serum and plasma [17, 18]. Thereafter, detailed analysis of conditioned medium collected from multiple lung cancer lines revealed a novel collection of candidate biomarkers [19]. More recently, subcellular fractionation and organelle isolation from freshly collected tissue specimens has enabled further candidate discovery, with verification achieved through multiple reaction monitoring (MRM) [20].

We have expanded on these approaches, broadening the scope of biomarker discovery by performing proteomic analyses across multiple types of specimens: freshly resected lung tissues, cancer cell lines and conditioned medium, enabling the discovery of a diverse collection of candidate markers. We have confirmed disease-enriched profiles for several of these candidates in sera collected from patients with early-stage disease. Moreover, a multi-marker model has been assembled which accurately distinguishing patients with NSCLC from smokers with no known malignancies. These studies suggest that the integration of multiple indirect discovery approaches may serve as a valuable means of identifying novel blood-based biomarkers that may be employed in the early detection and diagnosis of lung cancer.

## Results

### Tissue/cell-line based biomarker discovery

Three distinct LC/MS-based discovery programs were established to identify a diverse spectrum of candidate biomarkers which could serve as the basis for a blood-based immunoassay for detection or diagnosis of lung cancer. To enrich for markers destined to be found in the peripheral circulation of lung cancer patients, discovery focused on glycoproteins predicted to be located either at the cell membrane or secreted/shed from lung cancer cells. Cell-membrane discovery was performed in two distinct sample types: freshly resected tissue specimens (*n* = 13), and a collection of lung cancer cell lines (*n* = 17). The clinical specimens and cell lines studied provide broad coverage of tumor stage and prevalent histological cell types (Additional file 1: Table S1 and Additional file 2: Table S2). To focus discovery on differentially expressed candidate markers, peptide levels measured in surgically resected malignant samples were compared with adjacent normal tissue. Expression in lung cancer cell lines was analyzed relative to the non-cancerous immortalized lung epithelial cell line Beas-2B [21]. A third discovery program, which served to complement the cell-membrane analyses, resolved proteins secreted or shed from lung cancer cells into conditioned medium, in a subset of lines amenable to growth in serum-free conditions: A549, H1299, H358, H522, H2291, H520 and Calu-1.

Candidate lung cancer markers were prioritized based on MS data and predicted subcellular localization. To be selected, proteins had to: (i) be represented by multiple differentially expressed peptides (*n* > 1); (ii) be identified in multiple malignant samples (*n* > 1) and (iii) exhibit elevated expression in lung cancer specimens, with a cancer: control expression ratio of > 4.0. Candidate biomarkers were also prioritized based on secondary structure, with proteins predicted to be associated with either the cell membrane or secreted from the cell being selected; these two compartments are enriched with markers destined for the peripheral circulation. 179 candidate markers were identified which met these criteria (Additional file 3: Table S3).

**Fig. 1:**
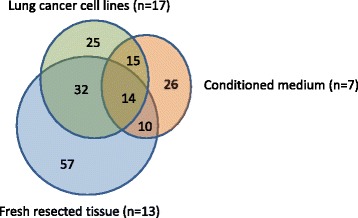
Venn diagram showing distribution of 179 candidate lung cancer biomarkers across 3 discovery platforms

Each of the cellular systems employed in these studies yielded a large number of candidate biomarkers: fresh resected tissues (*n* = 113), cell lines (*n* = 86) and conditioned medium (*n* = 65). While a small proportion of these biomarkers were identified in all three sample types (*n* = 14/179, 8 %), the majority were uniquely resolved in only one of the three cellular systems (*n* = 108/179, 60 %), highlighting the value of the multi-faceted approach (Fig. 1). 29 markers were discovered in both conditioned medium and cell-membrane preparations derived from lung cancer cell lines. Interestingly, a subset of these markers (*n* = 9/29, 31 %), was resolved in the same cell lines used for both membrane-bound and extracellular protein discovery. The overlapping detection of 9 markers in membrane-bound and secreted/shed preparations suggests multiple forms of these proteins may be expressed at elevated levels in NSCLC.

The Panther based classification system was used to categorize markers based on Protein Class and Pathway [22, 23]. Protein classes were defined for 141/179 (79 %) of the candidate markers evaluated. The most common classes reported were: receptors (14 %), cell adhesion molecules (14 %), hydrolases (13 %), defense/immunity proteins (10 %), proteases (9 %), enzyme modulators (8 %) and signaling molecules (8 %; Additional file 4: Figure S1). Further protein class analysis revealed similar profiles for biomarkers identified in the two cell-surface based discovery programs, resected tissue and cultured lung cancer cell lines (Additional file 5: Figure S2). Panther classification resolved protein categories for 91/113 (81 %) of the markers identified in tissues and 70/86 (81 %) of those found in cell lines. While some differences clearly exist, the most abundant protein classes (cell adhesion, defense/immunity, enzyme modulator, extracellular matrix, hydrolase, protease, receptor, signaling, transfer/carrier and transporter) were resolved in both tissues and cell lines. Panther-based pathway analysis also revealed many similarities between the two discovery platforms. Pathways commonly identified in resected tissues (integrin signaling, inflammation, gonadotropin releasing hormone receptor, Alzheimer disease-presenilin and plasminogen activating cascade) were also frequently found in the cell lines studied. Some differences were resolved between the two sources, including enrichment of blood-coagulation related proteins in the tissue based discovery system (22 %) relative to cell line studies (9 %; Additional file 6: Figure S3).

### Serum-based biomarker verification

ELISA analysis was undertaken to investigate whether the differential expression profiles observed in lung cancer tissues, cell lines and conditioned medium, would also be detected in the bloodstream of subjects with lung cancer. A small number of candidates were selected for serological characterization: CEA, MDK, MMP2, SLPI, TFPI and TIMP1 (Table 1). These biomarkers were selected in part due to the reagent availability, but also, with the exception of CEA, because they represented some of the more novel lung cancer markers identified, with few studies indicating elevated expression in the circulation of patients with early stage disease [24]. While all six markers had been shown to be present in plasma [25], they had not been resolved in other proteomic studies aimed at identifying differentially expressed lung cancer markers using alternative biological fluids: bronchial lavage [26, 27] sputum [28] or pleural fluid [29, 30], or in profiling experiments aimed at identifying markers associated with other common lung disorders: COPD [27], asthma [31] or tuberculosis [32].

**Table 1:** Candidate lung cancer biomarkers identified through MS discovery that were selected for serological characterization

Protein	Differentially expressed peptides	Number of samples where differentially expressed peptides observed			Differential expression (Lung cancer/control)	Predicted subcellular localization
		Tissues (*n* = 13)	Cell lines (*n* = 17)	Conditioned medium (*n* = 7)		
CEA (CEACAM5)	CETQNPVSAR	9	1		20.4	Cell Membrane
	TLTLFNVTRNDTASYK					
MDK	YNAQCQETIR			7	4.9	Secreted
	EGTCGAQTQR					
	FENWGACDGGTGTK					
	VTKPCTPK					
	YNAQCQETIRVTKPCTPK					
MMP2	ESCNLFVLK		1	3	21.8	Secreted
	TDKELAVQYLNTFYGCPK					
	ELAVQYLNTFYGCPK					
	CGNPDVANYNFFPR					
	YGFCPHEALFTMGGNAEGQPCK					
SLPI	SCVSPVKA	3			5.5	Secreted
	AGVCPPK					
	SCVSPVK					
	AGVCPPKK					
TFPI	ADDGPCK		1	3	10.0	Secreted
	QCEEFIYGGCEGNQNR					
	TTLQQEKPDFCFLEEDPGICR					
	YFYNNQTK					
TIMP1	FVYTPAMESVCGYFHR	11	1	4	34.6	Secreted
	HLACLPR					
	LQSGTHCLWTDQLLQGSEK					
	LQDGLLHITTCSFVAPWNSLSLAQR					
	FVGTPEVNQTTLYQRYEIK					
	AKFVGTPEVNQTTLYQR					
	FVGTPEVNQTTLYQR					
	SHNRSEEFLIAGK					
	EPGLCTWQSLR					

Differentially expressed peptides resolved for each marker are listed together with the number of samples (tissues, cell lines or conditioned medium) where peptides with elevated expression in lung cancer were identified. Median ratio represents the overall level of elevated expression combining levels observed for each differentially expressed peptide in disease samples relative to appropriate controls

With the goal of identifying markers to be used to screen for early-stage disease, or to guide diagnosis following CT-based detection, expression levels were determined in subjects with stage I NSCLC (*n* = 94), relative to normal smoker controls (*n* = 189; Table 2). In an effort to minimize selection of markers associated with pre-analytical variability, where differential expression profiles may be derived from serum sample collection procedures specific to any single clinical study site, subjects from two independent clinical studies were combined into a single testing set. The first study collected at CRCCC (Clinical Research Center of Cape Cod; West Yarmouth, MA), comprised patients with stage I NSCLC (*n* = 30) and healthy smoker controls (*n* = 99). The second cohort, collected at New York University (NYU) School of Medicine/Langone Medical Center, was selected from a high-risk population with a history of heavy tobacco usage. Serum samples were collected from patients with stage I NSCLC (*n* = 64) and healthy controls (*n* = 90).

**Table 2:** Demographic and clinical profiles of subjects tested with lung cancer biomarker candidates

	Serum verification/model training		Model testing	
	Controls	Cases	Controls	Cases
	(*N* = 189)	(*N* = 94)	(*N* = 50)	(*N* = 50)
Gender				
Male	109	33	28	28
Female	80	61	22	22
Age				
Mean (SD)	62.1 (11.8)	66.6 (9.6)	63.0 (6.4)	65.6 (6.7)
Smoking Pack Years				
Mean (SD)	37.6 (21.7)	43.9 (20.6)	54.3 (22.4)	54.2 (22.2)
Benign Nodules (n)			22	
Lesion Size (cm)				
Mean (range)			0.5 (0.2-1.2)	3.3 (0.8-12)
Stage				
I (%)		94 (100)		18 (36)
II (%)				6 (12)
III (%)				16 (32)
IV (%)				5 (10)
NA (%)				5 (10)
Histology				
Adenocarcinoma (%)		63 (67.0)		23 (46)
BAC (%)		4 (4.3)		
Large Cell (%)		6 (6.4)		
NSCLC (%)		4 (4.3)		6 (12)
Neuroendocrine (%)		2 (2.1)		
Small Cell (%)				4 (8)
Squamous Cell (%)		15 (16.0)		17 (34)

Levels of five of the six candidate biomarkers tested (CEA, MDK, MMP2, SLPI, TIMP1, TFPI) were significantly higher in serum from subjects with NSCLC than in controls (Table 3, Additional file 7: Figure S4), serving to support this indirect discovery approach. Three extensively characterized markers: CYFRA 21–1, SCC and OPN were also evaluated. These markers served as a reference in evaluating clinical accuracy of the MS-identified markers.

**Table 3:** Expression levels of biomarker candidates in serum collected from patients with NSCLC (*n* = 94) and healthy volunteer controls (*n* = 189)

	Control	Case	KS test (p-val)	AUC
	Median: ng/mL	Median: ng/mL		
	(Interquartile range)	(Interquartile range)		
CEA (CEACAM5)	1.65 (0.85-2.92)	2.68 (1.85-4.90)	<0.0001	0.706
MDK	0.15 (0.04-0.35)	0.43 (0.20-0.66)	<0.0001	0.714
MMP2	207 (184-234)	207 (171-254)	0.1472	0.492
SLPI	39.6 (34.8-46.2)	43.3 (35.5-54.5)	0.0036	0.595
TFPI	39.7 (25.6-55.1)	54.1 (29.1-70.3)	<0.0001	0.617
TIMP1	302 (269-346)	361 (306-440)	<0.0001	0.692
CYFRA 21-1	0.58 (0.00-1.05)	1.60 (0.91-3.00)	<0.0001	0.816
OPN	19.3 (10.0-31.0)	31.4 (16.7-52.4)	<0.0001	0.666
SCC	0.58 (0.34-0.93)	1.21 (0.55-1.70)	<0.0001	0.696

Median levels (ng/mL) and interquartile range are shown. The Kolmogorov–Smirnov test (KS test) was used to compare patient groups. Area under the curve (AUC) calculated from receiver operator characteristic (ROC) curve analysis. Markers in the upper section of the table (*n* = 6) represent proteins resolved through MS analysis. The lower section (*n* = 3) represents well-characterized lung cancer biomarkers

### Multi-marker model development and testing

The identification of multiple differentially expressed markers prompted the development of a multi-marker panel. Elastic net modeling [33] started with all 9 candidate markers (Table 3). The optimal value of the regularization parameter, as determined by bootstrap resampling, reduced the parameter estimate for SLPI to zero, while the remaining 8 markers: TFPI, MDK, OPN, MMP2, TIMP1, CEA, CYFRA 21–1 and SCC, which retained non-zero coefficients, were selected in the final model. In the training dataset (Table 2), this 8-marker model resolved lung cancer patients from smoker controls with 75 % sensitivity at 90 % specificity (AUC = 0.913). A bootstrap validation procedure confirmed clinical performance of the model, AUC = 0.903.

**Fig. 2:**
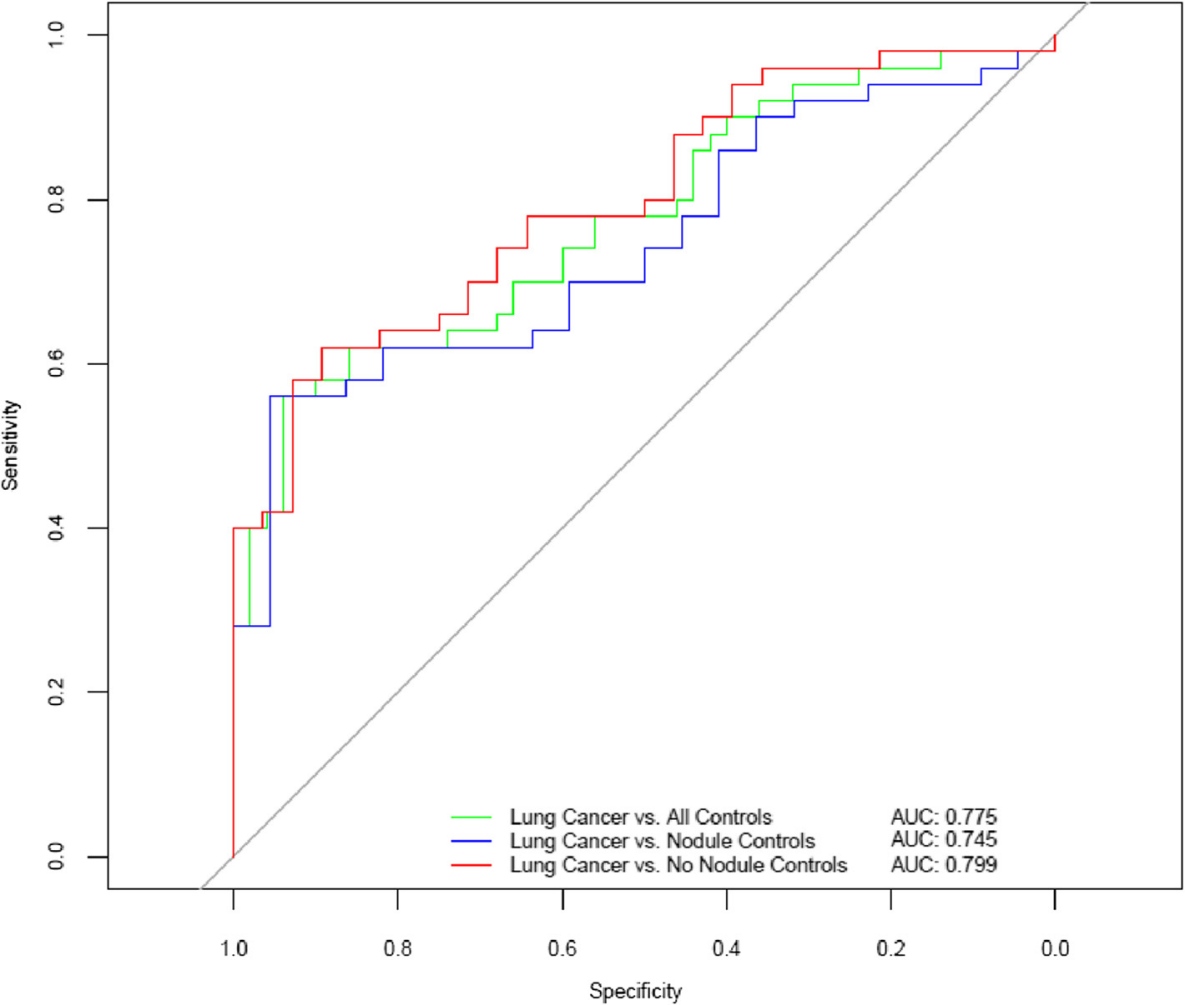
Multi-marker model resolves lung cancer cases from smoker controls. Receiver Operator Curves are plotted for all controls, nodule controls and no-nodule controls

The accuracy of the 8-marker model was tested in an independent study (Mayo Clinic). Controls (*n* = 50) were selected from the high risk control population evaluated in the Mayo CT-Screening Trial [34] and included subjects with pulmonary nodules (*n* = 22). Lung cancer cases were pre-operative surgical referrals (*n* = 50). Malignant lesions were significantly larger than screen detected benign nodules. Cases and controls were matched on age, gender and smoking history (Table 2). EDTA plasma samples were utilized in this study. Levels of all markers included in the model had been shown to be highly correlated in serum and EDTA plasma (Additional file 8: Table S4). The 8-marker model distinguished patients with malignant lesions from all smoker controls with an AUC = 0.775 (Fig. 2), accurately classifying control subjects with (AUC = 0.745) or without pulmonary nodules (AUC = 0.799).

While the 8-marker model was found to be substantially correlated with nodule size (r = 0.739; *p* < 0.0001), it was not associated with any of the other clinicopathological variables tested: age, sex, smoking history (unpublished data). Elevated expression of the multi-marker model was observed in tumors with a squamous cell histology, relative to adenocarcinoma cases (*p* = 0.019), driven in part by higher levels of CYFRA 21-1 (*p* < 0.0001) and OPN (*p* = 0.013) in squamous cell carcinomas (unpublished data).

## Discussion

LDCT screening of high-risk smokers has been shown to reduce lung cancer mortality by 20 %, relative to chest radiography. However, of the 24.2 % of participants with an abnormal screening test, the vast majority (96.4 %) were false positives for lung cancer. The low positive predictive value of LDCT results in (i) higher screening costs and (ii) unnecessary invasive procedures for benign disease. Non-invasive biomarkers are urgently needed to improve LDCT-based screening. Biomarkers could be used to refine the high-risk population, thus limiting the number of individuals being screened by LDCT. Alternatively, biomarkers could be employed following screening, to distinguish relatively rare malignant nodules from commonly found benign nodules. A number of novel blood-based markers have recently been characterized [7, 35], some of which have been evaluated in the form of multi-marker panels [15, 36–38]. However, to date, very few have been shown to add value to clinical variables already being employed in evaluating malignant risk [39].

Marker discovery in blood-based systems (serum and plasma) has been hampered by the complexity of these matrices and presence of multiple highly abundant proteins. Alternative “indirect” approaches have successfully been applied to: freshly resected clinical specimens, primary cultures, cell lines cultured *in vitro* and *in vivo* and conditioned medium, with collections of candidate biomarkers identified in each. However, as each of these studies has been performed in isolation, it has been difficult to evaluate the relative merits of each of these approaches. We report, for the first time, a discovery approach that combines multiple cellular systems: resected tissues, cultured cell lines and conditioned medium. In so doing, we have identified a number of markers commonly resolved across the platforms (Fig. 1). It is noteworthy that while significant overlap across the systems clearly exists, with similar signaling pathways apparently activated across the different discovery systems (Additional file 6: Figure S3), the majority of candidate markers were identified in only one of the three programs. Integrating discoveries from the three systems has not only served as a starting point to understand the relative merits of these distinct approaches, but has also produced a diverse pool of candidate markers for future validation.

All 179 candidate markers selected exhibited at least a four-fold increased level of expression in lung cancer samples relative to appropriate controls. While this 4.0X cut point provided a simple means of identifying the most differentially expressed biomarkers, additional approaches using different cut-points and possibly integrating key clinical variables such as histology and stage, will likely reveal a more extensive collection of candidates for future studies.

Analyses of the glycoproteins residing at the cell surface, in both tissues and cell lines, enabled discovery of cell-membrane markers that may be shed and released into the peripheral circulation. CEA (CEACAM5) provides an example of this type of cell-surface marker, as it is shed into the bloodstream and detected at elevated levels in a wide variety of malignant disorders [40]. While the molecular mechanism responsible for shedding remains unclear [41], CEA is a widely employed serum biomarker used in prognosis, staging and monitoring of colorectal cancer. In addition to CEA, several other cell-surface markers known to be shed into the circulation were identified in these studies including: MET (c-Met proto-oncogene product, hepatocyte growth factor receptor) [42], mesothelin [43], EPCAM [44], and ICAM-1 [45]. It is noteworthy that many additional cell-membrane markers, with similar secondary structures, were also resolved (Additional file 3: Table S3) and may serve as a valuable pool of candidate markers for future studies.

While CEA (CEACAM5) represents a well-characterized tumor biomarker, the association of other biomarkers with lung cancer varies considerably, with limited evidence of differential expression in early-stage disease. Increased activity of TFPI, a Kunitz-type serine protease inhibitor, has been reported in the circulation of patients with late-stage NSCLC [46]. MDK appears to play a role in both angiogenesis [47] and lung cancer metastasis [48]. Elevated levels of MDK, a heparin-binding growth factor, have been observed in serum collected from patients with a broad range of solid tumors, including lung cancer [49]. A number of tumor-stimulating functions have been demonstrated for TIMP1 [50, 51]. Elevated levels of this metallopeptidase have been observed in serum collected from subjects with late-stage NSCLC [52], with the highest levels reported in squamous cell carcinoma [53]. SLPI, a member of the Kazal superfamily of serine-proteinases, appears to play a role in tumor growth and metastasis [54–57]. Elevated levels of SLPI protein have been observed in the bloodstream of patients with NSCLC [24, 58]. While the matrix metalloproteinase MMP2 appears to play a role in lung cancer growth and migration [59–61], studies investigating levels of MMP2 in the bloodstream have reported inconsistent findings [62–64]. The diverse range of biological functions observed for markers identified in these studies are summarized in Table 4.

**Table 4:** Biological function of markers identified through mass spectroscopy that were selected for further validation

Gene name	Protein name	Protein ID (UniProtKB)	Function
CEA (CEACAM5)	Carcinoembryonic antigen-related cell adhesion molecule 5	P06731	Oncofetal glycoprotein not typically detected in adults. Plays role in cell adhesion and intracellular signaling [70, 71].
MDK	Midkine	P21741	Heparin binding cytokine promotes cellular transformation, angiogenesis and metastasis [47, 48].
MMP2	72 kDa type IV collagenase (Matrix metalloproteinase-2)	P08253	Degrades extracellular matrix, associated with tissue invasion, cell-induced angiogenesis and tumor growth and metastasis [59–61].
SLPI	Antileukoproteinase (Secretory leukocyte protease inhibitor)	P03973	Protects epithelial cells from serine proteases, promotes tumorigenic and metastatic pathways [54–57].
TFPI	Tissue factor pathway inhibitor	P10646	Serine protease inhibitor involved in clotting homeostasis [72].
TIMP1	Tissue inhibitor of metalloproteinase 1	P01033	Inactivates metalloproteinases by binding to zinc cofactor. Promotes proliferation and inhibits apoptosis [50, 51].

Our ELISA-based serological studies evaluated six candidate markers identified in these LC/MS analyses, along with three additional markers (CYFRA 21-1, SCC and OPN) that served as a benchmark of clinical accuracy. Two of these markers, CYFRA 21–1 and SCC, would not have been predicted to be resolved in these LC/MS studies as they lack an N-linked glycosylation site required for selection in the glycoprotein enrichment procedure. In contrast, two N-linked glycosylation sites are present in the mature form of the secreted protein OPN; as such, we would have expected peptides derived from this marker to be identified in these studies. It is unclear why OPN was not resolved; however it is possible that this marker may not have been differentially expressed in the samples analyzed, or that OPN-derived peptides may have been masked in the LC/MS separation.

The ability of the multi-marker model to distinguish lung cancer cases from control subjects with or without nodules indicates potential roles for the test, either as an adjunct to CT- screening, in determining risk of malignancy of pulmonary nodules, or in early lung cancer screening. Clearly additional studies are required to better characterize clinical performance of the current model and to evaluate of larger numbers of candidate biomarkers revealed in this study.

## Conclusions

Given the low PPV (4 %) of LDCT in screening the high risk population, there is a pressing need to discover non-invasive biomarkers to complement radiographic imaging in lung cancer screening and diagnosis.

We describe a broad-based discovery platform that has enabled the identification of a large, diverse collection of candidate lung cancer biomarkers. A subset of these markers identified “indirectly” in freshly resected tissue, cell lines and conditioned medium retained elevated cancer-associated expression profiles in the circulation of patients with early-stage disease. A multi-marker model was developed which accurately distinguished lung cancer cases from high risk smokers. This unique collection of markers should serve as a valuable resource for future clinical validation studies.

## Methods

### Tissue specimens

Freshly resected lung specimens (malignant lesions and normal adjacent tissue) were collected from 4 clinical sites using IRB-approved protocols: 1. Department of Pathology and Laboratory Medicine, University of Pennsylvania, Philadelphia, PA; 2. Division of Thoracic Surgery, University of Maryland Medical Center, Baltimore, MD; 3. Department of Cardiothoracic Surgery, George Washington University, Washington DC; 4. Asterand, Detroit, MI. To enrich for samples likely to produce strong MS signal, tissue specimens with a mass of at least 1 g were selected for this study. Single cell suspensions were prepared from each resected sample using a standard methodology before removal of red blood cells through addition of ACK lysis buffer [65]. Epithelial (EpCAM), leukocyte (CD45) content and cellular viability (PI exclusion) were determined by flow cytometry analysis (LSR I, BD Biosciences, San Jose, CA). Epithelial enrichment was undertaken using flow cytometry based cell sorting (EpCAM, Clone EBA-1, BD Biosciences). Samples yielding a minimum of 1x10^6^ viable epithelial cells were submitted for MS analysis.

### Cell lines and tissue culture

Lung cancer cell lines obtained from American Type Culture Collection (ATCC, Manassas, VA) or European Collection of Cell Cultures (ECACC, Salisbury, UK) were cultured in the appropriate media as recommended.

### Conditioned medium

Cell-lines were cultured to 70 % confluence, transferred to protein free media (293 SFM II, Invitrogen, Carlsbad, CA) for 72 h, after which cell debris was removed by centrifugation.

### Blood-based studies

Serum: A total of 283 subjects were evaluated in the verification/model training study, healthy smoker controls *n* = 189 and early stage NSCLC cases *n* = 94 (Table 2). Samples were collected during the period 2003–2008. Histological classification followed WHO guidelines recommended at the time of diagnosis.

Plasma: EDTA plasma was collected from 100 subjects in the model testing study, controls *n* = 50 and cases *n* = 50 (Table 2).

Serum: plasma correlations: Blood was drawn from subjects (*n* = 10) and collected into serum (red-cap) and EDTA plasma tubes on the same visit to the clinic (CRCCC). Concentrations levels were determined for all candidate biomarkers (*n* = 9). Marker levels were highly correlated (Additional file 8: Table S4).

For all studies, written informed consent was obtained from each subject. Samples were obtained prior to any treatment and were stored at −80 °C until use.

### Mass spectrometry

The discovery approach combined the enrichment of cell surface glycoproteins and secreted proteins with a decoupled (label-free), quantitative proteomics method. These programs focused discovery on markers containing short, tryptically-cleaved 5–25 amino acid peptides encoding either a cysteine residue or an N-linked glycosylation site, providing broad coverage of the proteome. A quantitative liquid LC/MS analysis of normal and tumor samples was used to identify peptide ions that were expressed at >4x levels in the cancer cells relative to the adjacent normal tissue. In cell line studies tumor lines were compared to Beas-2B. Subsequent MS/MS identification focused exclusively on peptides that had a relative change in abundance. To ensure data quality, manual inspection of each differentially expressed peptide ion was performed.

### Cell surface protein enrichment

Viable cells were incubated with 1 mM sodium periodate for 10 min to oxidize glycoproteins [66]. Oxidized glycoproteins were conjugated to hydrazide resin (Bio-Rad, Hercules, CA) at 4 °C overnight [67]. After washing sequentially with: 2 M NaCl, 2 % SDS, 200 mM propanolamine (0.1 M NaAcetate, pH 5.5), 40 % ethanol and 80 % ethanol; bound proteins were reduced with dithiothreitol, and alkylated with ICAT™ reagent (Life Technologies/Thermo Fisher Scientific, Applied Biosystems, Framingham, MA). Alkylated proteins were digested with trypsin and cysteine-containing peptides were captured using an avidin column (Life Technologies/Thermo Fisher Scientific). In addition to the cysteine-containing peptide fraction, peptides bound to the resin were also collected and analyzed. Release of peptides was achieved through PNGase-F digestion (New England BioLabs, Ipswich, MA.). While we found some overlap between the proteins identified in the two fractions, analysis of both the cysteine -containing fraction and the resin-bound fraction resulted in complementary coverage of the cell surface protein population.

### Conditioned medium preparation

Samples were lyophilized, reconstituted with deionized H_2_O, and dialyzed against 0.6 M Guanidine HCl, 10 mM Tris buffer, pH 8. Proteins were reduced with Tris (2-carboxyethyl) phosphine and alkylated with ICAT™ reagent (Life Technologies/Thermo Fisher Scientific). Following dialysis (0.1 M NH_4_Acetate), alkylated proteins were digested with trypsin. Cysteine-containing peptides were purified using an avidin column (Life Technologies/ Thermo Fisher Scientific).

### LC/MS analysis

Peptides, including standards used for mass calibration and retention time normalization, were separated and analyzed using methods of Kim et al. [68].

### Data alignment and expression analysis

Peptide ion peaks of LC/MS maps were aligned based on mass to charge ratio (*m/z*), retention time (Rt), and charge state (*z*). Retention time normalization was accomplished in two steps: a primary alignment using the internal standard peptides and a secondary fine tuning using all of the common features. Ion intensities were normalized across normal and tumor samples by minimizing the sum of the differences between the intensities of each of the ions and the mean intensity for that ion across all maps. Differentially expressed peptide ions were manually verified before LC-MS/MS-based peptide sequencing. Subcellular predictions determined by UniProt, release 2014_07 [69].

### Serum/plasma analyses

Enzyme-linked immunosorbent assay (ELISA) kits were obtained from a variety of commercial sources: Bio-Techne/R&D Systems, Minneapolis, MN (MMP2, OPN, SLPI, TFPI); Siemens Healthcare Diagnostics, Cambridge, MA (TIMP1); and IBL-America, Minneapolis, MN (CEA, CYFRA 21–1, MDK, SCC). Assays were performed following the manufacturers’ instructions. Plates were read on a Spectra Max M2 Microplate Reader (Molecular Devices, Sunnyvale, CA.).

### Model development

Logistic regression of lung cancer status on the 9 candidate markers (ng/mL) via elastic net regularization was employed to select a final set of markers and their associated parameter estimates. Elastic net regularization penalizes the parameter estimates (shrinks them toward zero) and performs variable subset selection by allowing sufficiently small parameter estimates to be reduced entirely to zero. Regularization of the parameter estimates tends to produce stable regression models with smaller prediction error than those that are not regularized. Bootstrap resampling (10,000 iterations), and maximization of the mean area under the ROC curve (AUC) for the out-of-bag samples, was used to select the optimal weight of the shrinkage penalty.

## Additional files

Additional file 1: Table S1. Demographics and clinical profiles for subjects selected for fresh tissue discovery study. Additional file 2: Table S2. Histology of lung cancer cell lines used for MS discovery study. Additional file 3: Table S3. Candidate lung cancer biomarkers (*n* = 179) identified through LC/MS analysis. Additional file 4: Figure S1. Panther-based classification of protein class for candidate lung cancer markers (*n* = 179). Additional file 5: Figure S2. Panther-based classification of protein class comparing lung cancer markers identified in tissues and cell lines. Additional file 6: Figure S3. Panther-based classification of protein pathways comparing lung cancer markers identified in tissues and cell lines. Additional file 7: Figure S4. Expression levels of biomarker candidates in serum collected from patients with NSCLC (*n* = 94) and healthy volunteer controls (*n* = 189). Additional file 8: Table S4. Correlation of marker levels in serum and plasma.

## Abbreviations

ACK: Ammonium-Chloride-Potassium
AUC: Area under curve
CEA: Carcinoembryonic antigen-related cell adhesion molecule 5
CYFRA 21–1: Cytokeratin-19 fragment
EDTA: Ethylenediaminetetraacetic acid
ELISA: Enzyme-linked immunosorbent assay
EpCAM: Epithelial cell adhesion molecule
ICAM: Intercellular Adhesion Molecule
IRB: Institutional Review Board
LC/MS: Liquid chromatography/mass spectrometry
LDCT: Low-dose computed tomography
MDK: Midkine
MET: c-Met proto-oncogene product
MRM: Multiple reaction monitoring
MMP2: Matrix metalloproteinase-2
NLST: National Lung Screening Trial
NSCLC: Non-small cell lung cancer
PI: Propidium Iodide
OPN: Osteopontin
PET-CT: Positron emission tomography–computed tomography
PPV: Positive predictive value
ROC: Receiver Operating Characteristic
SCC: Squamous cell carcinoma antigen
SLPI: Secretory Leukocyte Peptidase Inhibitor
TFPI: Tissue factor pathway inhibitor
TIMP1: Tissue inhibitor of metalloproteinase 1
USPSTF: US Preventive Services Task Force
