# Supplementary material for: Blood-based lung cancer biomarkers identified through proteomic discovery in cancer tissues, cell lines and conditioned medium
Source: Clin Proteomics. 2015 Jul 16;12(1):18. doi: 10.1186/s12014-015-9090-9 (PMC4537594; doi:10.1186/s12014-015-9090-9)
Supplement: Additional file 1: Table S1. — Demographics and clinical profiles for subjects selected for fresh tissue discovery study. [file 12014_2015_9090_MOESM1_ESM.pdf]

	Site	Gender	Age	Type	Stage
1	Penn	Male	59	Adenocarcinoma	IA
2	Maryland	Male	72	Squamous cell carcinoma	IA
3	Maryland	Female	54	Adenocarcinoma	IA
4	Penn	Female	68	Adenocarcinoma mixed	IB
5	Maryland	Female	66	Adenocarcinoma papillary	IB
6	Maryland	Female	72	Squamous cell carcinoma	IB
7	Penn	Female	47	Large cell carcinoma	IB
8	Penn	Male	65	Squamous cell carcinoma	IIB
9	Asterand	Female	52	Adenocarcinoma	IIB
10	George Washington	Male	82	Adenocarcinoma Bronchioloalveolar	IIIA
11	Asterand	Female	46	Adenosquamous carcinoma	IIIA
12	Maryland	Male	54	Adenocarcinoma	IIIA
13	Penn	Female	73	Adenocarcinoma mixed	IIIB

Supplementary Table 1: Demographics and clinical profiles for subjects selected for fresh tissue discovery study.
