# Supplementary material for: Blood-based lung cancer biomarkers identified through proteomic discovery in cancer tissues, cell lines and conditioned medium
Source: Clin Proteomics. 2015 Jul 16;12(1):18. doi: 10.1186/s12014-015-9090-9 (PMC4537594; doi:10.1186/s12014-015-9090-9)
Supplement: Additional file 2: Table S2. — Histology of lung cancer cell lines used for MS discovery study. [file 12014_2015_9090_MOESM2_ESM.pdf]

Lung Cancer Cell Line	Histology
A549	Adenocarcinoma
Calu-3	Adenocarcinoma
H2291	Adenocarcinoma
H23	Adenocarcinoma
H522	Adenocarcinoma
SK-LU-1	Adenocarcinoma
H1299	Adenocarcinoma
Calu-1	Epidermoid carcinoma
H460	Large cell carcinoma
H358	NSC bronchioloalveolar carcinoma
H727	NSC bronchus carcinoid
H441	Papillary adenocarcinoma
H69	Small cell lung carcinoma
H226	Squamous cell carcinoma
H520	Squamous cell carcinoma
SK-MES-1	Squamous cell carcinoma
SW-900	Squamous cell carcinoma

Supplementary Table 2: Histology of lung cancer cell lines selected for MS discovery study.
