# Supplementary material for: Blood-based lung cancer biomarkers identified through proteomic discovery in cancer tissues, cell lines and conditioned medium
Source: Clin Proteomics. 2015 Jul 16;12(1):18. doi: 10.1186/s12014-015-9090-9 (PMC4537594; doi:10.1186/s12014-015-9090-9)
Supplement: Additional file 3: Table S3. — Candidate lung cancer biomarkers (n = 179) identified through LC/MS analysis. [file 12014_2015_9090_MOESM3_ESM.pdf]

Supplementary Table S3: Candidate lung cancer biomarkers (n=179) identified through LC/MS analysis.										
Protein Name (Abreviated)	Gene Name	UniProt	Mass Spec						PANTHER Protein Class	PANTHER Pathway
			Tissue (n=13)	Cell Line (n=17)	Conditioned Medium (n=7)	# of Peptide Seqs	Median Ratio	Subcellular Localization		
Disintegrin and metalloproteinase domain-containing protein 10	ADAM10	O14672	2	4	2	9	11.6	Cell membrane		Alzheimer disease-amyloid secretase pathway->A disintegrase and metalloprotease 10;
Agrin	AGRN	O00468	0	0	3	9	19.5	Secreted	receptor;extracellular matrix linker protein	
Alpha-2-HS-glycoprotein	AHSG	P02765	2	2	9	5	4.1	Secreted	extracellular matrix glycoprotein;cysteine protease	
CD166 antigen	ALCAM	Q13740	5	7	1	15	9.0	Cell membrane	receptor;immunoglobulin superfamily cell adhesion	
Protein AMBP	AMBP	P02760	8	0	0	4	77.7	Secreted	serine protease inhibitor	
Angiogenin	ANG	P03950	0	0	2	2	4.8	Secreted	endoribonuclease;nuclease;hydrolase;enzyme modulator	
Aminopeptidase N	ANPEP	P15144	1	4	0	11	100.0	Cell membrane	metalloprotease;metalloprotease	
Amyloid-like protein 2	APLP2	Q06481	0	0	3	3	12.8	Cell membrane		
Beta-2-glycoprotein 1	APOH	P02749	7	0	0	8	6.5	Secreted	apolipoprotein;receptor;metalloprotease;serine	
Amyloid beta A4 protein	APP	P05067	0	0	5	4	7.0	Cell membrane	signaling molecule	Alzheimer disease-amyloid secretase pathway->Amyloid
Sodium/potassium-transporting ATPase subunit alpha-1	ATP1A1	P05023	4	4	0	10	5.7	Cell membrane	cation transporter;ion channel;hydrolase	
Sodium/potassium-transporting ATPase subunit beta-1	ATP1B1	P05026	2	10	0	10	11.7	Cell membrane	ATP synthase	
Sodium/potassium-transporting ATPase subunit beta-3	ATP1B3	P54709	5	6	0	6	9.6	Cell membrane	ATP synthase	
Beta-2-microglobulin	B2M	P61769	1	0	1	2	5.6	Secreted	major histocompatibility complex antigen	
Biglycan	BGN	P21810	3	1	0	3	15.0	Secreted	receptor;extracellular matrix protein	
Bone morphogenetic protein 1	BMP1	P13497	0	1	1	2	11.0	Secreted	transporter;apolipoprotein;membrane-bound signaling	TGF-beta signaling pathway->Transforming growth factor beta;
Basigin	BSG	P35613	6	5	0	6	7.3	Cell membrane	transmembrane receptor regulatory/adaptor protein	
ADP-ribosyl cyclase/cyclic ADP-ribose hydrolase 2	BST1	Q10588	3	2	0	5	66.7	Cell membrane	cyclase;glycosidase	
Bone marrow stromal antigen 2	BST2	Q10589	1	3	0	5	66.8	Cell membrane		
Complement C1q subcomponent subunit A	C1QA	P02745	2	0	0	3	8.2	Secreted	extracellular matrix structural protein;complement component	

Complement C3	C3	P01024	3	0	1	8	4.8	Secreted	cytokine;serine protease inhibitor;complement component	
C4b-binding protein alpha chain	C4BPA	P04003	11	0	0	11	6.1	Secreted	apolipoprotein;receptor;metalloprotease;serine	
Complement C5	C5	P01031	7	0	0	9	21.0	Secreted	cytokine;serine protease inhibitor;complement component	
Complement component C6	C6	P13671	6	0	0	13	20.6	Secreted	apolipoprotein;receptor;metalloprotease;serine	
Complement component C7	C7	P10643	9	0	0	19	14.6	Secreted	apolipoprotein;receptor;metalloprotease;serine	
Complement component C8 alpha chain	C8A	P07357	4	0	0	5	47.7	Secreted	apolipoprotein;receptor;metalloprotease;serine	
Complement component C8 beta chain	C8B	P07358	11	0	0	4	32.2	Secreted	apolipoprotein;receptor;metalloprotease;serine	
Complement component C8 gamma chain	C8G	P07360	5	0	0	3	11.3	Secreted	serine protease inhibitor	
Complement component C9	C9	P02748	12	0	0	10	36.2	Secreted	apolipoprotein;receptor;metalloprotease;serine	
Cathelicidin antimicrobial peptide	CAMP	P49913	7	0	0	6	16.6	Secreted	protease inhibitor;antibacterial response protein	
CD109 antigen	CD109	Q6YHK3	0	4	0	7	43.8	Cell membrane	cytokine;serine protease inhibitor;complement component	
Monocyte differentiation antigen CD14	CD14	P08571	2	0	0	4	9.4	Cell membrane		Toll receptor signaling pathway->CD14;
CD151 antigen	CD151	P48509	5	8	0	6	5.4	Cell membrane	membrane-bound signaling molecule;receptor;cell adhesion	
Scavenger receptor cysteine-rich type 1 protein M130	CD163	Q86VB7	2	0	0	4	10.8	Cell membrane	receptor;serine protease;oxidase;serine protease	
CD177 antigen	CD177	Q8N6Q3	3	0	0	5	26.5	Secreted		
Programmed cell death 1 ligand 1	CD274	Q9NZQ7	0	3	0	2	4.9	Cell membrane	membrane-bound signaling molecule;immunoglobulin	
CD276 antigen	CD276	Q5ZPR3	0	3	0	5	6.1	Cell membrane		
CD44 antigen	CD44	P16070	9	13	6	3	6.3	Cell membrane		Alzheimer disease-presenilin pathway->CD44 transmembrane fragment;
Membrane cofactor protein	CD46	P15529	3	4	0	5	8.0	Cell membrane	apolipoprotein;receptor;metalloprotease;serine	
Leukocyte surface antigen CD47	CD47	Q08722	0	3	0	3	7.2	Cell membrane	immunoglobulin receptor superfamily;guanyl-nucleotide	
Complement decay-accelerating factor	CD55	P08174	10	5	1	11	7.9	Cell membrane	apolipoprotein;receptor;metalloprotease;serine	
Lymphocyte function-associated antigen 3	CD58	P19256	0	2	0	2	7.8	Cell membrane		
CD59 glycoprotein	CD59	P13987	8	6	0	4	8.4	Cell membrane	membrane-bound signaling molecule	
CD97 antigen	CD97	P48960	0	2	0	4	13.2	Cell membrane	G-protein coupled receptor;antibacterial response	
CUB domain-containing protein 1	CDCP1	Q9H5V8	1	2	0	14	6.2	Cell membrane		

Cadherin-1	CDH1	P12830	0	2	2	3	5.4	Cell membrane	cell junction protein;cadherin	Alzheimer disease-presenilin pathway->E-cadherin C-terminal fragment; Alzheimer disease-presenilin pathway->E-cadherin transmembrane fragment; Wnt signaling pathway->Cadherin; Alzheimer disease-presenilin pathway->E-cadherin N-terminal fragment; Alzheimer disease-presenilin pathway->E-cadherin ;Alzheimer disease-presenilin pathway->E-cadherin intracellular fragment; Cadherin signaling pathway->Cadherin;
Cadherin-3	CDH3	P22223	0	1	2	2	8.6	Cell membrane	cell junction protein;cadherin	Alzheimer disease-presenilin pathway->E-cadherin C-terminal fragment; Alzheimer disease-presenilin pathway->E-cadherin transmembrane fragment; Wnt signaling pathway->Cadherin; Alzheimer disease-presenilin pathway->E-cadherin N-terminal fragment; Alzheimer disease-presenilin pathway->E-cadherin; Alzheimer disease-presenilin pathway->E-cadherin intracellular fragment; Cadherin signaling pathway->Cadherin;
Carcinoembryonic antigen-related cell adhesion molecule 1	CEACAM1	P13688	2	1	0	7	5.8	Cell membrane	immunoglobulin superfamily cell adhesion molecule	
Carcinoembryonic antigen-related cell adhesion molecule 5	CEACAM5	P06731	9	1	0	2	20.4	Cell membrane	immunoglobulin superfamily cell adhesion molecule	
Carcinoembryonic antigen-related cell adhesion molecule 8	CEACAM8	P31997	3	0	0	2	100.0	Cell membrane	immunoglobulin superfamily cell adhesion molecule	
Complement factor B	CFB	P00751	2	0	6	4	17.6	Secreted	apolipoprotein;receptor;metalloprotease;serine	
Complement factor H	CFH	P08603	13	0	3	20	16.0	Secreted	apolipoprotein;receptor;metalloprotease;serine	
Complement factor H-related protein 3	CFHR3	Q02985	2	0	0	2	25.5	Secreted	apolipoprotein;receptor;metalloprotease;serine	
Complement factor I	CFI IF	P05156	6	0	0	3	7.1	Secreted	serine protease;serine protease;annexin;calmodulin	
Secretogranin-1	CHGB	P05060	0	0	3	2	65.5	Secreted	peptide hormone	
Neural cell adhesion molecule L1-like protein	CHL1	O00533	0	1	1	5	32.7	Cell membrane	immunoglobulin receptor superfamily;protein	

Calsyntenin-1	CLSTN1	O94985	0	0	3	9	12.4	Cell membrane	cell adhesion molecule;calcium-binding protein	
Clusterin	CLU	P10909	11	2	1	10	12.4	Secreted		
Collagen alpha-1(I) chain	COL1A1	P02452	0	0	3	5	40.0	Secreted	transporter;surfactant;receptor;extracellular matrix structural	Integrin signalling pathway->Collagen;
Ceruloplasmin	CP	P00450	4	1	1	6	22.4	Secreted	transporter;apolipoprotein;membrane-bound signaling	
Connective tissue growth factor	CTGF	P29279	0	0	4	10	6.5	Secreted	growth factor	
Cathepsin D	CTSD	P07339	2	0	5	6	8.3	Secreted	aspartic protease;aspartic protease	
Cathepsin G	CTSG	P08311	10	0	0	5	44.9	Cell membrane	serine protease;serine protease	
C-X-C motif chemokine 5	CXCL5	P42830	0	0	2	2	120.0	Secreted	chemokine	
Protein CYR61	CYR61	O00622	0	0	4	7	9.6	Secreted	growth factor	
Dystroglycan	DAG1	Q14118	0	0	5	2	100.0	Secreted		
Neutrophil defensin 1	DEFA1	P59665	8	0	0	2	22.8	Secreted		
Dipeptidyl peptidase 4	DPP4	P27487	2	2	0	16	7.8	Cell membrane	serine protease;serine protease	
Desmoglein-2	DSG2	Q14126	0	2	0	4	12.3	Cell membrane	cell junction protein;cadherin	
EGF-containing fibulin-like extracellular matrix protein 1	EFEMP1	Q12805	0	0	3	5	18.3	Secreted		
Epidermal growth factor receptor	EGFR	P00533	2	3	0	25	11.5	Cell membrane		Gonadotropin releasing hormone receptor pathway->EGFR; EGF receptor signaling pathway->epidermal growth factor receptor; Cadherin signaling pathway->Epidermal growth factor receptor;
Ectonucleoside triphosphate diphosphohydrolase 2	ENTPD2	Q9Y5L3	2	0	0	4	100.0	Cell membrane	nucleotide phosphatase;nucleotide	
Epithelial cell adhesion molecule	EPCAM	P16422	2	5	0	6	6.1	Cell membrane	receptor	
Ephrin type-B receptor 2	EPHB2	P29323	0	2	0	6	13.3	Cell membrane		Angiogenesis->Ephrin Receptor;
Prothrombin	F2	P00734	9	0	0	14	12.2	Secreted	serine protease;serine protease	Blood coagulation->Thrombin; Blood coagulation->Prothrombin;
Proteinase-activated receptor 1	F2R	P25116	3	0	0	2	50.0	Cell membrane	G-protein coupled receptor	Angiogenesis->Protease Activated Receptor; Blood coagulation->Protease-activated receptor1;
Protein FAM3C	FAM3C	Q92520	0	0	3	2	21.3	Secreted		

Immunoglobulin alpha Fc receptor	FCAR	P24071	2	0	0	2	56.4	Cell membrane	membrane-bound signaling molecule;immunoglobulin	
Fetuin-B	FETUB	Q9UGM5	2	0	0	2	10.9	Secreted		
Fibronectin	FN1	P02751	4	1	4	21	7.4	Secreted	signaling molecule	Integrin signalling pathway->Fibronectin;
Folate receptor beta	FOLR2	P14207	3	0	0	2	50.0	Cell membrane		
Vitamin D-binding protein	GC	P02774	7	0	0	12	16.3	Secreted		Vitamin D metabolism and pathway->Vitamin D binding
Glypican-1	GPC1	P35052	0	4	0	6	8.3	Cell membrane	extracellular matrix glycoprotein;cell adhesion	
Glypican-4	GPC4	O75487	2	1	2	4	9.6	Cell membrane	extracellular matrix glycoprotein;cell adhesion	
Probable G-protein coupled receptor 116	GPR116	Q8IZF2	0	2	0	3	37.7	Cell membrane	G-protein coupled receptor;antibacterial response	
Retinoic acid-induced protein 3	GPRC5A	Q8NFJ5	0	2	0	2	18.2	Cell membrane	G-protein coupled receptor	
Granulins	GRN	P28799	0	3	0	4	11.0	Secreted		
HLA class II histocompatibility antigen, DR beta 4 chain	HLA-DRB4	P13762	3	0	0	2	54.8	Cell membrane	major histocompatibility complex antigen	
Hemopexin	HPX	P02790	8	0	0	6	9.9	Secreted	transfer/carrier protein;metalloprotease;metallop	
Histidine-rich glycoprotein	HRG	P04196	2	0	0	2	11.4	Secreted		
Basement membrane-specific heparan sulfate proteoglycan core	HSPG2	P98160	0	1	4	24	10.1	Secreted	receptor;extracellular matrix linker protein	
Intercellular adhesion molecule 1	ICAM1	P05362	1	7	1	13	6.4	Cell membrane	signaling molecule;immunoglobulin	
Intercellular adhesion molecule 3	ICAM3	P32942	3	0	0	5	16.4	Cell membrane	signaling molecule;immunoglobulin	
Insulin-like growth factor-binding protein 2	IGFBP2	P18065	0	0	2	8	30.2	Secreted		
Insulin-like growth factor-binding protein 3	IGFBP3	P17936	0	0	8	7	8.3	Secreted		p53 pathway->Insulin-like growth factor-binding protein 3;
Insulin-like growth factor-binding protein 4	IGFBP4	P22692	0	0	7	7	16.4	Secreted		
Insulin-like growth factor-binding protein 7	IGFBP7	Q16270	0	0	2	4	6.1	Secreted		
Immunoglobulin superfamily member 8	IGSF8	Q969P0	0	1	1	4	35.4	Cell membrane		
Integrin alpha-2	ITGA2	P17301	3	3	0	17	11.2	Cell membrane	cell adhesion molecule	Integrin signalling pathway->Integrin alpha; Inflammation mediated by chemokine and cytokine signaling pathway->ExtraCellular matrix protein;
Integrin alpha-3	ITGA3	P26006	5	8	1	16	6.0	Cell membrane	cell adhesion molecule	Integrin signalling pathway->Integrin alpha;

Integrin alpha-5	ITGA5	P08648	0	3	0	8	8.4	Cell membrane	cell adhesion molecule	Integrin signalling pathway->Integrin alpha;
Integrin alpha-6	ITGA6	P23229	0	4	0	13	5.3	Cell membrane	cell adhesion molecule	Integrin signalling pathway->Integrin alpha;
Integrin alpha-M	ITGAM	P11215	9	0	0	12	23.5	Cell membrane	cell adhesion molecule	Integrin signalling pathway->Integrin alpha; Inflammation mediated by chemokine and cytokine signaling pathway->Integrin;
Integrin alpha-V	ITGAV	P06756	7	5	0	15	6.7	Cell membrane	cell adhesion molecule	Integrin signalling pathway->Integrin alpha;
Integrin beta-1	ITGB1	P05556	7	7	5	14	5.6	Cell membrane	receptor;extracellular matrix glycoprotein;cell adhesion molecule	Integrin signalling pathway->Integrin beta; Gonadotropin releasing hormone receptor pathway->alpha-beta integrin dimer; Inflammation mediated by chemokine and cytokine signaling pathway->Integrin;
Integrin beta-2	ITGB2	P05107	9	0	0	20	15.2	Cell membrane	receptor;extracellular matrix glycoprotein;cell adhesion molecule	Integrin signalling pathway->Integrin beta; Inflammation mediated by chemokine and cytokine signaling pathway->Integrin;
Integrin beta-4	ITGB4	P16144	4	4	0	15	4.2	Cell membrane	receptor;extracellular matrix glycoprotein;cell adhesion	Integrin signalling pathway->Integrin beta;
Integrin beta-6	ITGB6	P18564	0	2	0	6	16.8	Cell membrane	receptor;extracellular matrix glycoprotein;cell adhesion	Integrin signalling pathway->Integrin beta;
Inter-alpha-trypsin inhibitor heavy chain H2	ITIH2	P19823	4	0	0	2	11.8	Secreted	serine protease inhibitor	
Junction plakoglobin	JUP	P14923	2	1	0	9	27.5	Cell membrane	storage protein;signaling molecule;cytoskeletal protein;cell	Alzheimer disease-presenilin pathway->gamma-catenin;
Kallikrein-6	KLK6	Q92876	0	0	4	5	36.2	Secreted	serine protease;serine protease	
Plasma kallikrein	KLKB1	P03952	5	0	0	5	60.1	Secreted	serine protease;serine protease	Blood coagulation->kallikrein;;Blood coagulation->Prekallikrein;
Kininogen-1	KNG1	P01042	12	0	0	9	13.8	Secreted		Blood coagulation->High molecular weight kininogen;;Blood coagulation->Bradykinin;;Blood coagulation->Kininogen;
Laminin subunit alpha-5	LAMA5	O15230	0	2	1	14	18.8	Secreted	receptor;extracellular matrix linker protein	Integrin signalling pathway->Laminin;
Neutrophil gelatinase-associated lipocalin	LCN2	P80188	8	0	4	4	20.3	Secreted	transfer/carrier protein;isomerase	
Galectin-3-binding protein	LGALS3BP	Q08380	6	2	3	9	8.0	Secreted	receptor;serine protease;oxidase;serine protease	

Lysyl oxidase homolog 2	LOXL2	Q9Y4K0	0	0	3	3	13.9	Secreted	receptor;serine protease;oxidase;serine protease	
Lactotransferrin	LTF	P02788	8	0	8	38	36.5	Secreted	transfer/carrier protein;serine protease;serine protease	
Lumican	LUM	P51884	5	0	0	4	8.2	Secreted	receptor;extracellular matrix protein	
Cell surface glycoprotein MUC18	MCAM	P43121	3	5	0	10	4.9	Cell membrane	receptor;immunoglobulin superfamily cell adhesion	
Midkine	MDK	P21741	0	0	7	5	4.9	Secreted	cytokine	
Hepatocyte growth factor receptor	MET	P08581	0	1	1	8	17.6	Cell membrane		
Melanotransferrin	MFI2	P08582	4	2	0	15	8.4	Cell membrane	transfer/carrier protein	
Neprilysin	MME	P08473	0	2	0	8	69.2	Cell membrane	metalloprotease;metalloprotease	
Matrix metalloproteinase-2	MMP2	P08253	0	1	3	5	21.8	Secreted	metalloprotease;metalloprotease; extracellular matrix protein	Alzheimer disease-presenilin pathway->Matrix metalloprotease;
Mesothelin	MSLN	Q13421	0	2	0	8	67.6	Cell membrane	extracellular matrix glycoprotein	
Nicotinamide phosphoribosyltransferase	NAMPT	P43490	6	0	1	2	7.6	Secreted	cytokine	
Neural cell adhesion molecule 1	NCAM1	P13591	1	1	0	5	50.1	Cell membrane	immunoglobulin receptor superfamily;protein	
Epididymal secretory protein E1	NPC2	P61916	3	0	8	5	7.7	Secreted		
Neuroplastin	NPTN	Q9Y639	0	3	0	4	5.4	Cell membrane	transmembrane receptor regulatory/adaptor protein	
Neuronal cell adhesion molecule	NRCAM	Q92823	0	2	0	10	45.7	Cell membrane	immunoglobulin receptor superfamily;protein	
5'-nucleotidase	NT5E	P21589	0	6	0	7	8.7	Cell membrane	nucleotide phosphatase;nucleotide phosphatase;phosphodiesterase	Pyrimidine Metabolism->5'-Nucleotidase;;Purine metabolism->5'-Nucleotidase;
Olfactomedin-4	OLFM4	Q6UX06	3	0	0	7	12.5	Secreted	structural protein;receptor	
Alpha-1-acid glycoprotein 2	ORM2	P19652	4	0	0	2	11.2	Secreted		
N-acetylmuramoyl-L-alanine amidase	PGLYRP2	Q96PD5	5	0	0	3	21.6	Secreted	signaling molecule;defense/immunity	
Urokinase-type plasminogen activator	PLAU	P00749	0	1	4	7	9.1	Secreted	serine protease;serine protease	Blood coagulation->urokinase plasminogen activator; Plasminogen activating cascade->urokinase type plasminogen activator; Plasminogen activating cascade->pro-urokinase type plasminogen activator;

Urokinase plasminogen activator surface receptor	PLAUR	Q03405	0	1	2	8	7.5	Cell membrane		Blood coagulation->urokinase-type plasminogen activator receptor; Plasminogen activating cascade->urokinase type plasminogen activator receptor;
Plasminogen	PLG	P00747	13	0	0	22	17.0	Secreted	serine protease;serine protease	Blood coagulation->Plasminogen; Plasminogen activating cascade->Plasminogen; Blood coagulation->Plasmin; Plasminogen activating cascade->Plasmin;
Myelin proteolipid protein	PLP1	P60201	3	0	0	2	50.0	Cell membrane	myelin protein	
Phospholipid transfer protein	PLTP	P55058	4	0	0	6	15.2	Secreted	transfer/carrier protein;antibacterial response	
Plexin-B2	PLXNB2	O15031	0	4	0	11	6.3	Cell membrane	tyrosine protein kinase receptor;signaling	
Podocalyxin	PODXL	O00592	5	2	0	4	35.5	Cell membrane		
Serum paraoxonase/arylesterase 1	PON1	P27169	5	0	0	2	46.8	Secreted		
Bone marrow proteoglycan	PRG2	P13727	4	0	0	5	7.6	Secreted	extracellular matrix structural protein;antibacterial response	
Vitamin K-dependent protein S	PROS1	P07225	2	0	0	3	12.9	Secreted		Blood coagulation->Protein S;
Serine protease 23	PRSS23	O95084	1	0	2	6	39.4	Secreted		
Inactive tyrosine-protein kinase 7	PTK7	Q13308	0	4	0	14	11.2	Cell membrane	non-receptor tyrosine protein kinase;tyrosine protein kinase	
Receptor-type tyrosine-protein phosphatase C	PTPRC	P08575	6	0	0	9	8.0	Cell membrane	receptor;protein phosphatase;protein phosphatase	T cell activation->CD45; JAK/STAT signaling pathway->protein-tyrosine phosphatase; B cell activation->CD45;
Eosinophil cationic protein	RNASE3	P12724	6	0	0	6	12.5	Secreted	endoribonuclease;nuclease;hydrolase;enzyme modulator	
Alpha-1-antichymotrypsin	SERPINA3	P01011	2	0	0	4	5.7	Secreted	serine protease inhibitor	
Antithrombin-III	SERPINC1	P01008	3	0	0	4	20.4	Secreted	serine protease inhibitor	Blood coagulation->Antithrombin III;
Glia-derived nexin	SERPINE2	P07093	0	1	1	4	6.0	Secreted	serine protease inhibitor	
Alpha-2-antiplasmin	SERPINF2	P08697	10	0	0	3	41.2	Secreted	serine protease inhibitor	Blood coagulation->alpha2-antiplasmin; Plasminogen activating cascade->alpha2 antiplasmin;

Plasma protease C1 inhibitor	SERPING1	P05155	7	0	0	4	8.4	Secreted	serine protease inhibitor	
Neutral amino acid transporter B(0)	SLC1A5	Q15758	0	4	0	5	12.2	Cell membrane	cation transporter	
Solute carrier family 23 member 2	SLC23A2	Q9UGH3	0	2	0	2	11.0	Cell membrane	transporter	
4F2 cell-surface antigen heavy chain	SLC3A2	P08195	5	7	0	7	5.4	Cell membrane	amylase	
Solute carrier family 43 member 3	SLC43A3	Q8NBI5	0	2	0	2	8.1	Cell membrane		
Antileukoproteinase	SLPI	P03973	3	0	0	4	5.5	Secreted	serine protease inhibitor	
Sortilin	SORT1	Q99523	3	0	0	3	100.0	Cell membrane	transporter;receptor	
Kunitz-type protease inhibitor 1	SPINT1	O43278	0	1	3	10	8.2	Secreted	serine protease inhibitor	
Kunitz-type protease inhibitor 2	SPINT2	O43291	0	1	5	2	4.3	Cell membrane	serine protease inhibitor	
Sushi repeat-containing protein SRPX2	SRPX2	O60687	0	0	2	3	8.0	Secreted	apolipoprotein;receptor;metalloprotease;serine	
Stanniocalcin-2	STC2	O76061	0	0	2	3	8.1	Secreted	peptide hormone	
Synaptophysin	SYP	P08247	2	0	0	2	60.2	Cell membrane	membrane trafficking regulatory protein	Synaptic_vesicle_trafficking->Synaptophysin;
Serotransferrin	TF	P02787	11	1	0	31	8.1	Secreted	transfer/carrier protein	
Tissue factor pathway inhibitor	TFPI	P10646	0	1	3	4	10.0	Secreted	serine protease inhibitor	Blood coagulation->Tissue Factor Pathway Inhibitor;
Tissue factor pathway inhibitor 2	TFPI2	P48307	0	0	6	6	4.8	Secreted	serine protease inhibitor	
Transferrin receptor protein 1	TFRC	P02786	3	4	0	7	9.1	Cell membrane	receptor	
Transforming growth factor-beta-induced protein ig-h3	TGFBI	Q15582	0	0	5	6	17.5	Secreted	signaling molecule;cell adhesion molecule	
Thy-1 membrane glycoprotein	THY1	P04216	4	3	0	4	4.4	Cell membrane		
Metalloproteinase inhibitor 1	TIMP1	P01033	11	1	4	9	34.6	Secreted	metalloprotease inhibitor	
Metalloproteinase inhibitor 2	TIMP2	P16035	0	0	3	3	8.8	Secreted	metalloprotease inhibitor	
Metalloproteinase inhibitor 3	TIMP3	P35625	6	0	0	4	33.0	Secreted	metalloprotease inhibitor	
Transmembrane 9 superfamily member 3	TM9SF3	Q9HD45	5	1	0	5	4.4	Cell membrane	transporter	

Transmembrane protein 33	TMEM33	P57088	6	0	0	3	15.1	Cell membrane		
Versican core protein	VCAN	P13611	0	0	3	3	11.9	Secreted	extracellular matrix glycoprotein	
Voltage-dependent anion-selective channel protein 1	VDAC1	P21796	1	3	0	2	10.9	Cell membrane	anion channel;voltage-gated ion channel	
Vitronectin	VTN	P04004	15	1	0	8	49.4	Secreted	cell adhesion molecule	

The number of differentially expressed peptides resolved for each marker are listed together with the number of samples (tissues, cell lines or conditioned medium) where peptides with elevated expression in lung cancer were identified.
In cases where signal from control sample(s) was below detection threshold, a cancer:control ratio=100 was employed.
Median ratio represents overall level of elevated expression combining levels observed for each differentially expressed peptide in disease samples relative to appropriate controls.
Subcellular location predicition (UniProt).
Panther based prediction of protein class and pathway.
