# Supplementary material for: Blood-based lung cancer biomarkers identified through proteomic discovery in cancer tissues, cell lines and conditioned medium
Source: Clin Proteomics. 2015 Jul 16;12(1):18. doi: 10.1186/s12014-015-9090-9 (PMC4537594; doi:10.1186/s12014-015-9090-9)
Supplement: Additional file 4: Figure S1. — Panther-based classification of protein class for candidate lung cancer markers (n = 179). [file 12014_2015_9090_MOESM4_ESM.pdf]

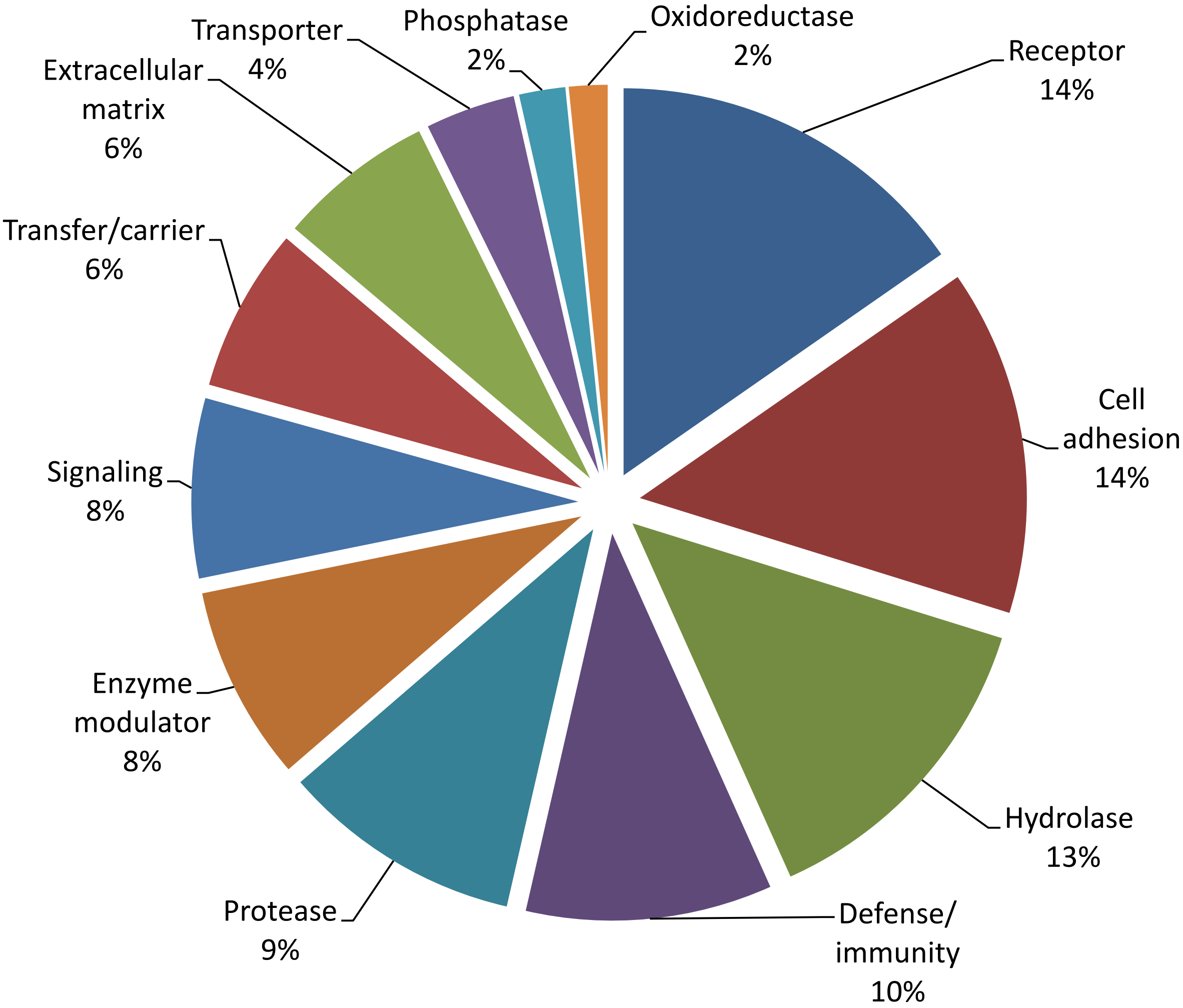

Supplementary Figure 1: Panther-based classification of protein class for candidate lung cancer markers (n=179). The 12 most common categories are plotted, each representing at least 2% of the total number of proteins.
