# Supplementary material for: Blood-based lung cancer biomarkers identified through proteomic discovery in cancer tissues, cell lines and conditioned medium
Source: Clin Proteomics. 2015 Jul 16;12(1):18. doi: 10.1186/s12014-015-9090-9 (PMC4537594; doi:10.1186/s12014-015-9090-9)
Supplement: Additional file 5: Figure S2. — Panther-based classification of protein class comparing lung cancer markers identified in tissues and cell lines. [file 12014_2015_9090_MOESM5_ESM.pdf]

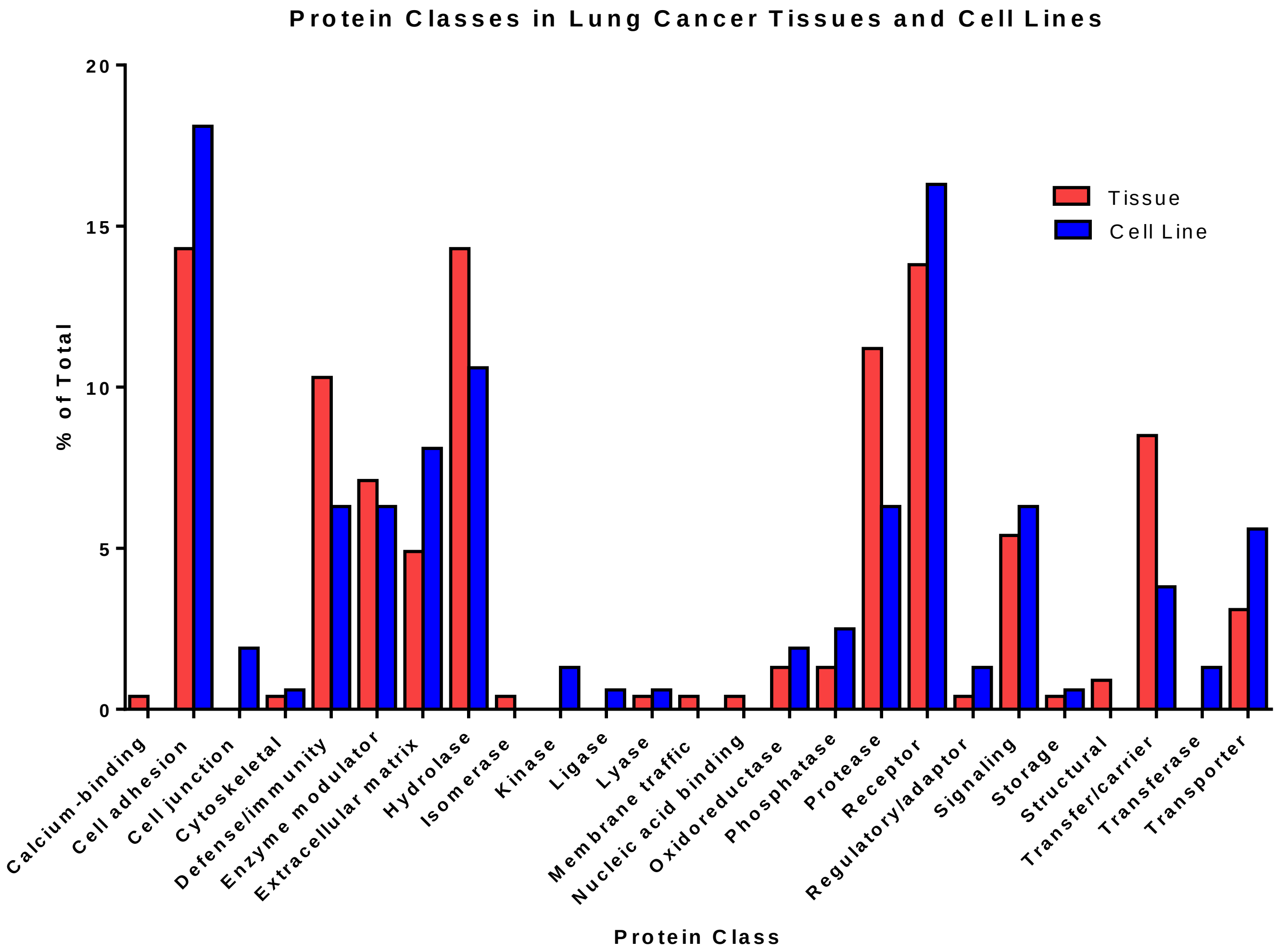

Supplementary Figure 2: Panther-based classification of protein class comparing lung cancer markers identified in tissues and cell lines.
