# Supplementary material for: Blood-based lung cancer biomarkers identified through proteomic discovery in cancer tissues, cell lines and conditioned medium
Source: Clin Proteomics. 2015 Jul 16;12(1):18. doi: 10.1186/s12014-015-9090-9 (PMC4537594; doi:10.1186/s12014-015-9090-9)
Supplement: Additional file 7: Figure S4. — Expression levels of biomarker candidates in serum collected from patients with NSCLC (n = 94) and healthy volunteer controls (n = 189). [file 12014_2015_9090_MOESM7_ESM.pdf]

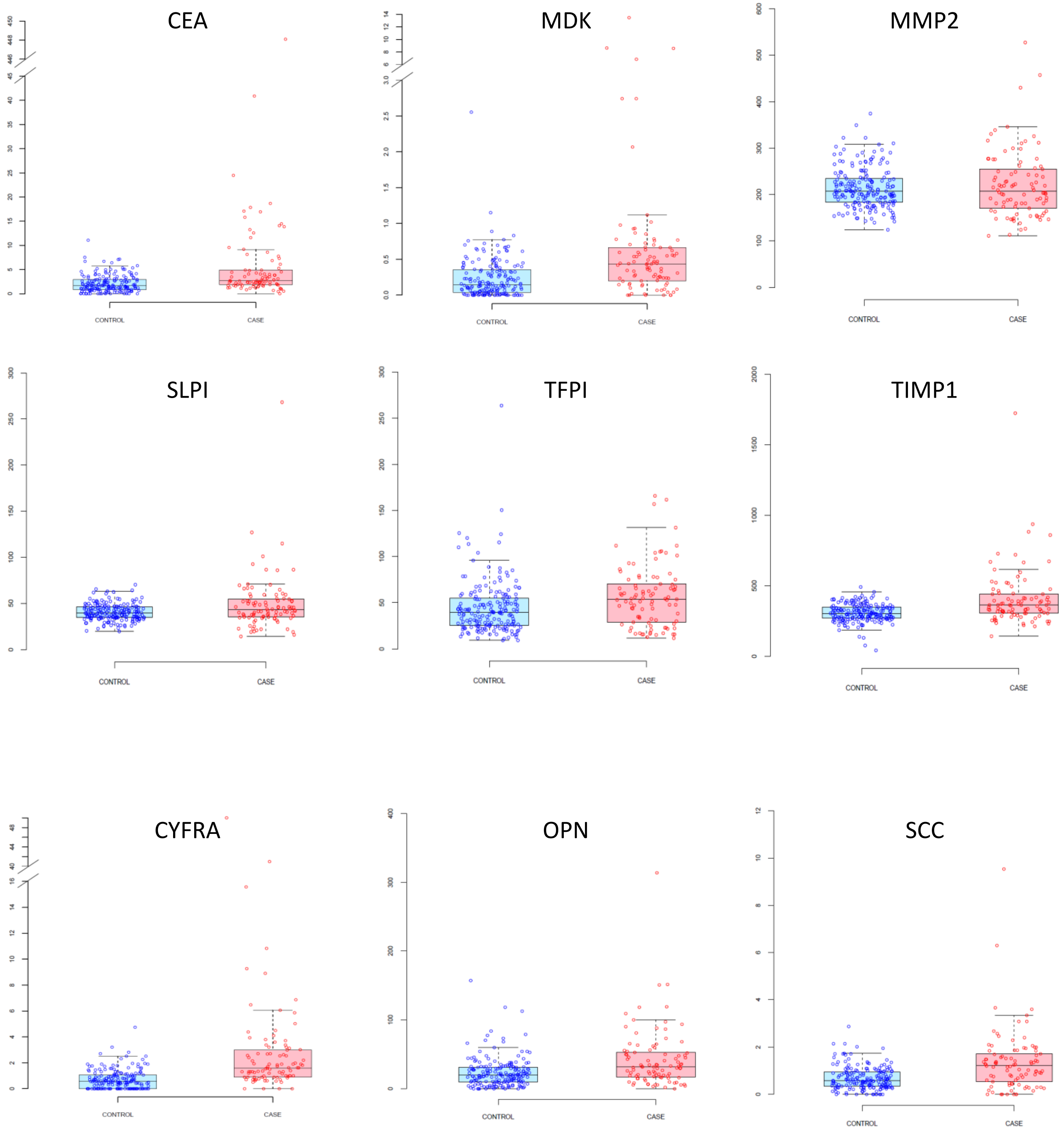

Supplementary Figure 4: Expression levels (ng/mL) of biomarker candidates in serum collected from patients with NSCLC (n=94) and healthy volunteer controls (n=189) are shown. Median level and interquartile range are plotted. Markers in the upper section (n=6) represent proteins resolved through MS analysis. The lower section (n=3) represents well-characterized lung cancer biomarkers.
