# Supplementary material for: Blood-based lung cancer biomarkers identified through proteomic discovery in cancer tissues, cell lines and conditioned medium
Source: Clin Proteomics. 2015 Jul 16;12(1):18. doi: 10.1186/s12014-015-9090-9 (PMC4537594; doi:10.1186/s12014-015-9090-9)
Supplement: Additional file 8: Table S4. — Correlation of marker levels in serum and plasma. [file 12014_2015_9090_MOESM8_ESM.pdf]

	r	95% Confidence Interval	R^2	P value
CEA	0.958	0.827 to 0.990	0.918	< 0.0001
CYFRA 21-1	0.962	0.844 to 0.991	0.926	< 0.0001
SCC	0.989	0.953 to 0.997	0.978	< 0.0001
MDK	0.939	0.691 to 0.989	0.881	0.0006
TFPI	0.716	0.158 to 0.928	0.513	0.0198
OPN	0.902	0.630 to 0.977	0.814	0.0004
MMP-2	0.950	0.796 to 0.988	0.902	< 0.0001
SLPI	0.877	0.552 to 0.971	0.769	0.0009
TIMP-1	0.821	0.396 to 0.956	0.674	0.0036

Supplementary Table 4: Correlation of marker levels in serum and plasma.
